# Different degree of cytokinemia and T-cell activation according to serum IL-6 levels in critical COVID-19

**DOI:** 10.3389/fimmu.2023.1110874

**Published:** 2023-04-04

**Authors:** Chan Mi Lee, Minji Kim, Chang Kyung Kang, Pyoeng Gyun Choe, Nam Joong Kim, Hyeeun Bang, Taeeun Cho, Hyun Mu Shin, Hang-Rae Kim, Wan Beom Park, Myoung-don Oh

**Affiliations:** ^1^ Department of Internal Medicine, Seoul National University College of Medicine, Seoul, Republic of Korea; ^2^ Department of Biomedical Sciences, Seoul National University College of Medicine, Seoul, Republic of Korea; ^3^ Department of Anatomy & Cell Biology and Biomedical Sciences, Seoul National University College of Medicine, Seoul, Republic of Korea; ^4^ BK21 FOUR Biomedical Science Project, Seoul National University College of Medicine, Seoul, Republic of Korea; ^5^ Research and development team 2, Molecular Diagnostics Division, Quantamatrix Inc., Seoul, Republic of Korea; ^6^ Wide River Institute of Immunology, Seoul National University, Hongcheon, Republic of Korea; ^7^ Medical Research Institute, Seoul National University College of Medicine, Seoul, Republic of Korea

**Keywords:** immune response, cytokine, IL-6, T cell, critical COVID-19

## Abstract

**Introduction:**

Tocilizumab, a humanized anti-interleukin-6 receptor (IL-6R) antibody, is recommended for the treatment of severe to critical coronavirus diseases 2019 (COVID-19). However, there were conflicting results on the efficacy of tocilizumab. Therefore, we hypothesized that the differences in tocilizumab efficacy may stem from the different immune responses of critical COVID-19 patients. In this study, we described two groups of immunologically distinct COVID-19 patients, based on their IL-6 response.

**Methods:**

We prospectively enrolled critical COVID-19 patients, requiring oxygen support with a high flow nasal cannula or a mechanical ventilator, and analyzed their serial samples. An enzyme-linked immunosorbent assay and flow cytometry were used to evaluate the cytokine kinetics and cellular immune responses, respectively.

**Results:**

A total of nine patients with critical COVID-19 were included. The high (*n* = 5) and low IL-6 (*n* = 4) groups were distinguished by their peak serum IL-6 levels, using 400 pg/mL as the cut-off value. Although the difference of flow cytometric data did not reach the level of statistical significance, the levels of pro-inflammatory cytokines and the frequencies of intermediate monocytes (CD14^+^CD16^+^), IFN-γ^+^ CD4^+^ or CD8^+^ T cells, and HLA-DR^+^PD-1^+^ CD4^+^ T cells were higher in the high IL-6 group than in the low IL-6 group.

**Conclusion:**

There were distinctive two groups of critical COVID-19 according to serum IL-6 levels having different degrees of cytokinemia and T-cell responses. Our results indicate that the use of immune modulators should be more tailored in patients with critical COVID-19.

## Introduction

About 5% of patients with coronavirus disease 2019 (COVID-19) experience critical illness, characterized by respiratory failure, septic shock, and multiorgan failure (1, 2). T-cell hyperactivation (3), cytokine storm (4), and expansion of monocyte subpopulation (5) have been suggested as typical immunological features of severe COVID-19; thus immune modulators such as corticosteroid, baricitinib, or tocilizumab are used for the treatment of severe to critical COVID-19 (6).

Although the National Institutes of Health guidelines recommend the use of tocilizumab, a humanized monoclonal antibody against the interleukin-6 receptor (IL-6R), for the treatment of critical COVID-19 (7), there have been conflicting results relating to the efficacy of this drug (8–10). This raises the question of whether patients with critical COVID-19 differ in their immune responses. If different immunopathologies, rather than viral pathological mechanisms, contribute to disease severity in patients with critical COVID-19, more tailored anti-inflammatory strategies could be pursued. Therefore, we aimed to describe and classify immune responses especially according to IL-6 response, among patients with critical COVID-19.

## Method

### Study population and design

In this study, adult (≥ 18 years old) patients with critical acute respiratory syndrome coronavirus 2 (SARS-CoV-2) infection, hospitalized to the Seoul National University Hospital between August to December 2021, were prospectively enrolled. All SARS-CoV-2 infection was laboratory-confirmed by quantitative real-time reverse transcription polymerase chain reaction (qRT-PCR). Critical COVID-19 was defined as patients requiring respiratory support such as high flow nasal cannula (HFNC) oxygen therapy or mechanical ventilation.

Serial blood and nasopharyngeal swab samples were collected around the onset of critical illness. Clinical data, including demographics, underlying comorbidities, COVID-19 vaccination status, disease severity, COVID-19 specific treatment, duration of isolation, and clinical outcome were collected. The levels of C-reactive protein (CRP) were also retrospectively collected.

This study was approved by the Institutional Review Board (IRB) of Seoul National University Hospital (IRB no. 2104-181-1215). All participants provided written informed consent in accordance with the Declaration of Helsinki.

### Measurement of anti-S1 immunoglobulin G

Serum anti-SARS-CoV-2 S1 domain (*i.e*., S1) immunoglobulin (Ig) G titers were semi-quantitatively measured using the anti-SARS-CoV-2 enzyme-linked immunosorbent assay (ELISA) IgG kit (Euroimmun, Lübeck, Germany), according to the manufacturer’s instructions. The optical density (O.D. _450 nm_) ratios were interpreted as follows: ≥ 1.1, positive; ≥ 0.8 to < 1.1, borderline; < 0.8, negative.

### Quantification of serum cytokines

The levels of IL-6, monocyte chemoattractant protein (MCP)-1, interferon-gamma (IFN-γ), and tumor necrosis factor-alpha (TNF-α) were measured in serum samples using the Human IL-6 ELISA Kit II (#550799, BD Biosciences, San Jose, CA, USA), Human MCP-1 ELISA Kit (#559017, BD Biosciences), Human IFN-γ Quantikine ELISA Kit (R&D Systems, Inc., Minneapolis, MN, USA), and Human TNF ELISA Kit (#550610, BD Biosciences) respectively, according to the manufacturer’s instruction.

A previous study set the threshold of IL-6 > 406 pg/mL for predicting intensive care unit (ICU) mortality of COVID-19 patients (11). Therefore, in our study, patients were classified as the high IL-6 group if their peak IL-6 levels were over 400 pg/mL; otherwise, they were classified as the low IL-6 group.

### Detection of SARS-CoV-2 RNA using qRT-PCR

Viral RNA was extracted from nasopharyngeal swab samples using the QIAamp Viral RNA Mini kit (Qiagen, Hilden, Germany), according to the manufacturer’s instructions. Briefly, 140 μL of nasopharyngeal swabs was mixed with 560 μL of lysis buffer and incubated for 10 min at 37°C. The supernatant containing the viral RNA was purified, and the extracted RNA was eluted in 50 μL of elution buffer and stored at –70°C until use. qRT-PCR for detection of SARS-CoV-2 was performed using the QPLEX™ COVID-19 Test (QMCOVID02, QuantaMatrix Inc., Seoul, Republic of Korea). A 20 μL of PCR mixture contained 10 μL of 2× One-step Premix, 5 μL of Oligo Mix, 5 μL of extracted RNA, and primer and probe sequences targeting the *RdRp* gene of SARS-CoV-2. Thermal cycling was performed at 25°C for 10 min for the uracil-DNA glycosylase incubation step, at 52°C for 5 min for the reverse transcription step, followed by 95°C for 10 sec, 5 cycles of pre-amplification (95°C for 10 sec and 55°C for 30 sec), and 35 cycles of the PCR reaction (95°C for 10 sec and 55°C for 30 sec) using the CFX-96 *In Vitro* Diagnostics Real-Time PCR System (Bio-Rad Laboratories, Inc., Hercules, CA, USA). A cycle threshold (Ct) value higher than 32 was defined as negative.

### Peripheral blood mononuclear cells isolation

Human peripheral blood mononuclear cells (PBMCs) were isolated from heparinized blood by Ficoll–Plaque PLUS (1.077 g/mL; GE Healthcare Life Sciences, Piscataway, NJ, USA) density gradient centrifugation. Purified PBMCs were cryopreserved in 50% fetal bovine serum (FBS), 10% dimethyl sulfoxide, and 40% RPMI-1640 (all reagents from Thermo Fisher Scientific, Waltham, MA, USA) at 5 × 10^6^ cells/mL and thawed prior to use (12).

### Flow cytometric analysis

After thawing the PBMCs, cells were pelleted by centrifugation and resuspended in phosphate-buffered saline (pH 7.4) supplemented with 1% FBS at a final density of 1 × 10^7^ cells/mL. Cells were stained using brilliant violet 711 (BV711)–anti-human CD3 (clone, UCHT1), brilliant ultra violet 805 (BUV805)–anti-human CD14 (clone, MØpq), and allophycocyanin-H7–anti-human CD16 (clone, 3G8) antibodies (Abs) (all from BD Biosciences). Brilliant Stain Buffer (BD Biosciences) was added to each sample.

For the staining of activation markers and cytokines, the PBMCs (at 1 × 10^7^ cells/mL) were stimulated with 50 ng/mL of phorbol 12-myristate 13-acetate and 1 μg/mL of ionomycin (both reagents from Sigma-Aldrich, St. Louis, MO, USA) and then, 1 h later, treated with BD Golgistop™ (Monensin, BD Biosciences) for an additional 3 h. BV605–anti-human CD4 Ab (clone, OKT-4; Biolegend, San Diego, CA, USA) was applied concomitantly during stimulation. Stimulated cells were stained with BV711–anti-human CD3 (clone, UCHT1), BUV496–anti-human CD8 (clone, RPA-T8), BUV395–anti-human PD-1 (clone, MIH4), and phycoerythrin (PE)-cyanine-5–anti-human HLA-DR (clone, G46-6) Abs (all from BD Biosciences). After fixation and permeabilization with the Cytofix/Cytoperm kit (BD Biosciences), cells were incubated with the PE–anti-human IFN-γ Ab (clone, B27; BD Biosciences). Samples were acquired on a BD LSRII flow cytometer (BD Biosciences) and analyzed using FlowJo software version 10.7.1 (TreeStar, Ashland, OR, USA). The percentages of target cell populations in unstimulated specimens were subtracted from those in stimulated specimens (13).

### Statistical analyses

The experimental data were presented as the median with interquartile range (IQR). The Mann–Whitney *U* test was used to compare continuous variables. Statistical analyses were conducted using SPSS Statistics, version 26.0 (IBM Corp., Armonk, NY, USA). *P*-values < 0.05 were considered as a measure of statistical significance. GraphPad Prism 9 (GraphPad Software, La Jolla, CA, USA) was used to generate graphs.

## Results

### Study participants and kinetics of serum SARS-CoV-2 antibody

A total of nine patients with critical COVID-19 were enrolled during the study period (**Table 1**). The high IL-6 group included five (55.6%) patients and the low IL-6 group included four (44.4%) patients (**Table 1**). Among the nine patients, seven patients (77.8%) were male and the median (range) age was 70 (58–84). All patients had multiple underlying comorbidities, such as hypertension (6/9, 66.7%) or diabetes mellitus (5/9, 55.6%) and were fully vaccinated with the AZD1222 (5/9, 55.6%) or BNT162b2 (4/9, 44.4%); the diagnosis of COVID-19 was made more than 14 days after their second vaccine dose (14). All patients were treated with remdesivir and steroids, and four (44.4%) patients were treated with tocilizumab. Two (2/5, 40%) patients from the high IL-6 group and one (1/4, 25%) patients from the low IL-6 group required mechanical ventilation, although all patients enrolled in this study made a full recovery.

**Table 1:** Clinical information of study participants.

IL-6 Group	Pt #	Age	Sex	Underlying disease	Vaccine status	Vaccine type	Severity of COVID-19	Treatment for COVID-19	Days of isolation	Outcome
High	1	76	Female	HTN, DM, DL	Fully vaccinated	BNT162b2	High flow	Remdesivir Steroid	17	Recovery
High	2	58	Male	LC, DM	Fully vaccinated	BNT162b2	Mechanical ventilator	Remdesivir Steroid Tocilizumab	18	Recovery
High	3	79	Female	Atrial fibrillation, aortic stenosis	Fully vaccinated	BNT162b2	Mechanical ventilator	Remdesivir Steroid Tocilizumab	30	Recovery
High	4	63	Male	HTN, DM	Fully vaccinated	AZD1222	High flow	Remdesivir Steroid Baricitinib	20	Recovery
High	5	68	Male	DM, HTN, DL	Fully vaccinated	AZD1222	High flow	Remdesivir Steroid Tocilizumab	21	Recovery
Low	6	60	Male	Angina, HTN, DL	Fully vaccinated	AZD1222	High flow	Remdesivir Steroid Baricitinib	10	Recovery
Low	7	84	Male	HTN, BPH	Fully vaccinated	BNT162b2	Mechanical ventilator	Remdesivir Steroid Tocilizumab	25	Recovery
Low	8	65	Male	HTN, DM, peripheral artery disease	Fully vaccinated	AZD1222	High flow	Remdesivir Steroid Baricitinib	7	Recovery
Low	9	70	Male	Chronic DVT	Fully vaccinated	AZD1222	High flow	Remdesivir Steroid Baricitinib	11	Recovery

IL-6, interleukin-6; HTN, hypertension; DM, diabetes mellitus; DL, dyslipidemia; LC, liver cirrhosis; BPH, benign prostate hyperplasia; DVT, deep vein thrombosis.

Fully vaccinated refers to individuals who were diagnosed with COVID-19 more than 14 days after their second COVID-19 vaccine dose.

The kinetics of viral load, the anti-S1 IgG and CRP levels, and the use of immune modulators in the high and low IL-6 groups are presented in **Supplementary Figure S1A, B**, respectively. The day of HFNC oxygen therapy initiation was designated as day 0. We found that the viral load decreased with time and all patients acquired anti-S1 IgG during their clinical course. In most patients, the level of CRP peaked near the onset of critical illness. Serial serum samples were used to evaluate cytokine levels and representative PBMC samples were used for the characterization of the cellular immune response.

### Cytokine levels and kinetics vary according to IL-6 response

**Figure 1:**
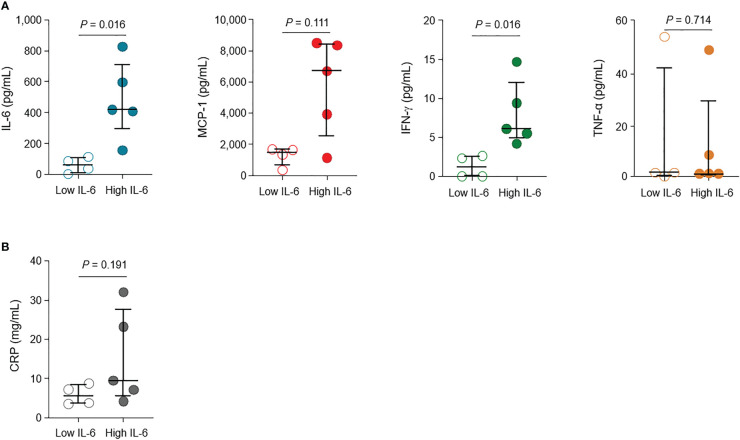
Levels of cytokines and inflammatory marker in patients with critical COVID-19. **(A)** The levels of interleukin (IL)-6, monocyte chemoattractant protein (MCP)-1, interferon (IFN)-γ, and tumor necrosis factor (TNF)-α in the high IL-6 (*n* = 5) and low IL-6 (*n* = 4) groups. **(B)** Levels of C-reactive protein (CRP) in the high IL-6 and low IL-6 groups. Vertical and horizontal lines indicate the median with the interquartile range. The cytokine and CRP levels at the closest point to the onset of critical illness (within 0–5 days of high flow nasal cannula oxygen therapy initiation) are shown.

The levels of cytokines (IL-6, MCP-1, IFN-γ, and TNF-α) and CRP according to the groups are shown in **Figures 1A**, **B**, respectively. The cytokine levels measured within 0−5 days of initiating high flow nasal cannula oxygen therapy, at the closest point to the onset of critical illness, have been presented. The specific time point during the clinical course is indicated with pink arrows in **Supplementary Figure S1A, B**, allowing for a simultaneous evaluation with the administration of immune modulators. The levels of IL-6 and IFN-γ were significantly higher in the high IL-6 group than in the low IL-6 group (median [IQR], 419 pg/mL [283–710] vs. 63 pg/mL [11–107], *P* = 0.016; 6.11 pg/mL [4.84–12.04] vs. 1.16 pg/mL [0.00–2.56], *P* = 0.016, respectively). While not statistically significant, the levels of MCP-1 and CRP were also higher in the high IL-6 group than in the low IL-6 group (median [IQR], 6,700 pg/mL [2,535–8,417] vs. 1,492 pg/mL [615–1,684], *P* = 0.111; 9.49 mg/dL [5.69–27.61] vs. 5.49 mg/dL [3.60–8.40], *P* = 0.191, respectively). The levels of TNF-α did not show significant difference between the high and low IL-6 groups (median [IQR], 0.08 pg/mL [0.00–27.53] vs. 1.36 pg/mL [0.32–41.89], *P* = 0.714).

The kinetics of cytokine levels in individual patients are presented in **Supplementary Figure S1C**, according to the days from critical illness. Overall, the low IL-6 group had lower levels of IL-6, MCP-1, and IFN-γ than the high IL-6 group.

### Monocyte subpopulation and cellular immune response against SARS-CoV-2

**Figure 2:**
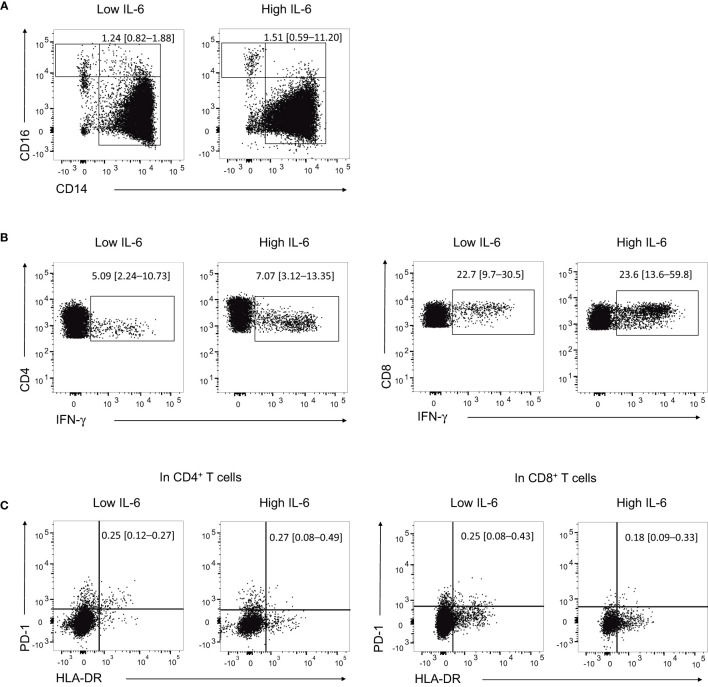
Proportion of monocyte subpopulation and expression levels of cytokine and activation markers in T cells. **(A)** Representative flow cytometry dot plots showing the identification of intermediate monocytes (CD14^+^CD16^+^) in the high IL-6 (*n* = 5) and low IL-6 (*n* = 4) groups. **(B)** Representative flow cytometry dot plots showing the identification of IFN-γ^+^ CD4^+^ T cells and CD8^+^ T cells in the high IL-6 and low IL-6 groups. **(C)** Representative flow cytometry dot plots showing the identification of HLA-DR^+^PD-1^+^ CD4^+^ T cells and CD8^+^ T cells in the high IL-6 and low IL-6 groups. Numbers indicate population frequencies as the median with interquartile range.

To provide a temporal context for the flow cytometric analysis samples in relation to the clinical course and immune modulator treatment, we marked the respective time points with green arrows in **Supplementary Figure S1A, B**. The high IL-6 group showed a higher frequency of intermediate monocytes (CD14+CD16+) compared to the low IL-6 group, although the difference was not statistically significant (%, median [IQR], 1.51 [0.59–11.20] vs. 1.24 [0.82–1.88], *P* = 0.730; **Figure 2A**). On the other hand, the high IL-6 group had higher frequencies of IFN-γ+ in CD4+ and CD8+ T cells compared to the low IL-6 group (%, median [IQR], 7.07 [3.12–13.35] vs. 5.09 [2.24–10.73], *P* = 0.730; 23.6 [13.6–59.8] vs. 22.7 [9.7–30.5], *P* = 0.730; **Figure 2B**).

Regarding activated T cells based on the expression of HLA-DR and PD-1, the high IL-6 group had a slightly higher proportion of HLA-DR+PD-1+ CD4+ T cells compared to the low IL-6 group, although the difference was not statistically significant (%, median [IQR], 0.27 [0.08–0.49] vs. 0.25 [0.12–0.27], *P* = 0.492). In contrast, the high IL-6 group had a lower proportion of HLA-DR+PD-1+ CD8+ T cells compared to the low IL-6 group, but the difference was not statistically significant either (%, median [IQR], 0.18 [0.09–0.33] vs. 0.25 [0.08–0.43], *P* = 0.730; **Figure 2C**). The flow cytometric data from all samples are presented in **Supplementary Figure S2**.

## Discussion

In this study, we found that patients with critical COVID-19 can be classified by two distinctive groups in terms of cytokinemia and T-cell activation levels, according to their levels of serum IL-6. The high IL-6 group had higher levels of pro-inflammatory cytokines and cellular immune responses than the low IL-6 group. The hypercytokinemia and T-cell hyperactivation of the high IL-6 group suggest that immunological features could differ among patients with critical COVID-19, despite similar clinical characteristics.

Since IL-6 is considered as an important immunological factor in severe/critical COVID-19 (15, 16), clinical trials of tocilizumab for the treatment of severe COVID-19 have been conducted. For instance, the RECOVERY trial found that tocilizumab improved the clinical outcomes of COVID-19 patients with hypoxia (oxygen saturation < 92%) and CRP levels ≥ 7.5 mg/dL (17). However, other studies did not show the efficacy of tocilizumab in patients with severe COVID-19 (9, 10). One retrospective study also reported that the early use of tocilizumab was associated with improvement of oxygenation in patients with high IL-6 levels (18). These conflicting results led us to perform the present study, to elucidate whether certain immunological features, and especially the IL-6 response, differed among patients with critical COVID-19.

Results from the early phase of SARS-CoV-2 pandemic suggested that the host immune response, such as levels of cytokinemia, might play an important role in the pathogenesis of severe COVID-19 (19). For instance, the plasma levels of several cytokines, including MCP-1 and TNF-α, were shown to be higher in ICU than non-ICU COVID-19 patients (16). Another study demonstrated that the levels of several cytokines (e.g., IL-6, IL-10, and MCP-1) positively correlated with COVID-19 severity; moreover, MCP-1 was correlated with days on mechanical ventilation (20). A previous study has shown that increased levels of IL-6 are strongly associated with disease severity at admission and the need for ICU care in COVID-19 patients, regardless of age (21). In our present study, we aimed to demonstrate that cytokine levels could vary significantly among critically ill COVID-19 patients, using serum IL-6 levels as a surrogate marker. Our findings suggest that IL-6 could be an important marker for classifying COVID-19 patients, even after the general population has been vaccinated, as all patients in our study were vaccinated.

In addition, we detected higher frequencies of intermediate monocytes (CD14^+^CD16^+^) in the high IL-6 group than in the low IL-6 group. Intermediate monocytes expand in patients with systemic infection and secrete cytokines, such as TNF-α, IL-1β, and IL-6, implying their role in pathogen defense (22). A previous study suggested that CD14^+^CD16^+^ monocytes may exhibit dysregulated production of IL-6 (23). Another study also reported that monocyte-mediated hypercytokinemia is prominent in critical COVID-19 (24). Moreover, the frequencies of intermediate monocytes were higher in COVID-19 patients admitted to ICU (5). The higher levels of cytokines in the high IL-6 group might therefore correlate with the higher frequency of intermediate monocytes.

We showed that the high IL-6 group exhibited stronger cellular immune responses than the low IL-6 group, based on the expression of cytokines and T-cell activation markers. Although T-cell hyperactivation is a key immunological feature of severe COVID-19 (3, 25), the results of the present study suggest that the magnitude of T-cell responses could vary even in clinically similar critical COVID-19. Corticosteroid might attenuate T-cell responses as well as cytokinemia in critical COVID-19, however, we could find distinctive two groups of critical COVID-19 in this study.

In conclusion, we identified that patients with critical COVID-19 could be divided into two groups with different degrees of cytokinemia and T-cell responses, according to their serum IL-6 levels. Our results suggest that a more tailored use of immune modulators should be sought in the treatment of critical COVID-19.

## Data availability statement

The original contributions presented in the study are included in the article/**Supplementary Material**. Further inquiries can be directed to the corresponding authors.

## Ethics statement

The studies involving human participants were reviewed and approved by the Institutional Review Board (IRB) of Seoul National University Hospital. The patients/participants provided their written informed consent to participate in this study.

## Author contributions

CL, MK, CK, HS, H-RK, and WP conceived and designed the study. CL, CK, PC, NK, WP, and M-DO collected the samples. CL, MK, CK, HB, TC, HS, H-RK, and WP performed data analysis. NK, HS, H-RK, WP, and M-DO revised and edited the final manuscript. All authors contributed to the article and approved the submitted version.
