# Supplementary material for: Different degree of cytokinemia and T-cell activation according to serum IL-6 levels in critical COVID-19
Source: Front Immunol. 2023 Apr 4;14:1110874. doi: 10.3389/fimmu.2023.1110874 (PMC10110916; doi:10.3389/fimmu.2023.1110874)
Supplement: Supplementary file 1 [file DataSheet_1.docx]

**Supplement Contents**

Supplementary Figure S1. ------------------------------------------------------------------- page 2

Supplementary Figure S2. ------------------------------------------------------------------- page 3-4

**Supplement Figure Legends**

**Supplementary Figure S1. Kinetics of viral load, anti-S1 IgG, C-reactive protein and cytokines.** (A) The kinetics of viral load, anti-S1 IgG, and C-reactive protein (CRP) in the high interleukin (IL)-6 group (*n* = 5). (B) The kinetics of viral load, anti-S1 IgG, and CRP in the low IL-6 group (*n* = 4). (C) Kinetics of IL-6, monocyte chemoattractant protein (MCP)-1, interferon (IFN)-γ, and tumor necrosis factor (TNF)-α in the high IL-6 and low IL-6 groups. The day of high flow nasal cannula oxygen therapy initiation was designated as day 0. The time points of Figure 1A and Figure 2 were marked with pink and green arrows, respectively.

**Supplementary Figure S2. Monocyte subpopulation and expression levels of cytokine and activation markers in T cells.** (A) Dot plots showing the identification of intermediate monocytes (CD14^+^CD16^+^) for all samples in both high IL-6 (*n* = 5) and low IL-6 (*n* = 4) groups. (B) Dot plots showing the identification of IFN-γ^+^ CD4^+^ T cells and CD8^+^ T cells for all samples in both high IL-6 and low IL-6 groups. (C) Dot plots showing the identification of HLA-DR^+^PD-1^+^ CD4^+^ T cells and CD8^+^ T cells for all samples in both high IL-6 and low IL-6 groups. Numerical values indicate population frequencies.

**
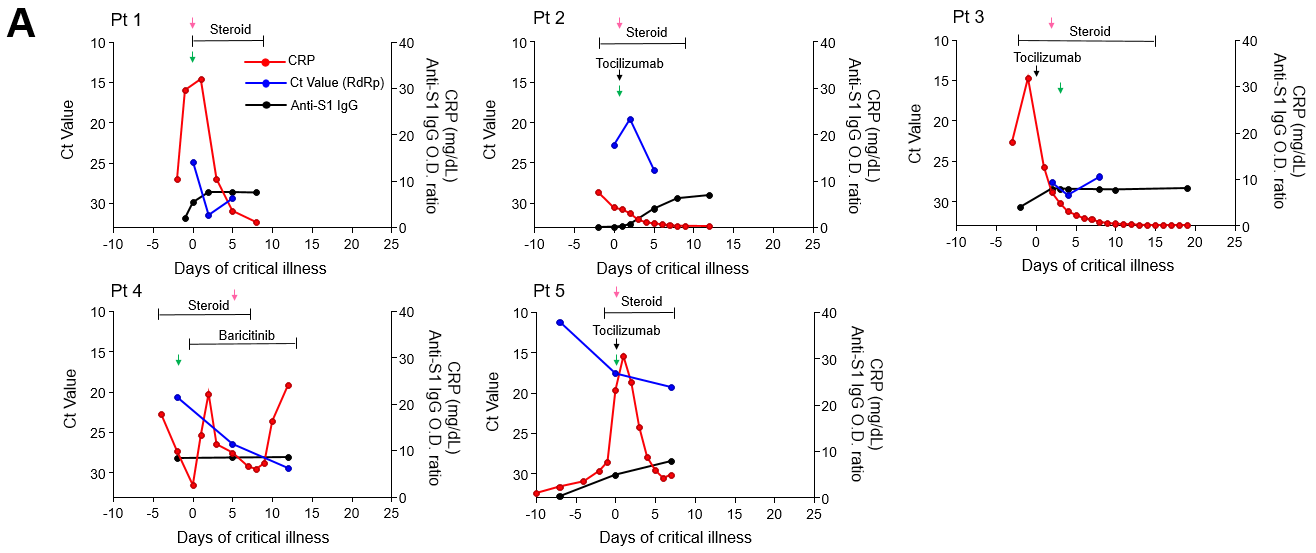
**

**
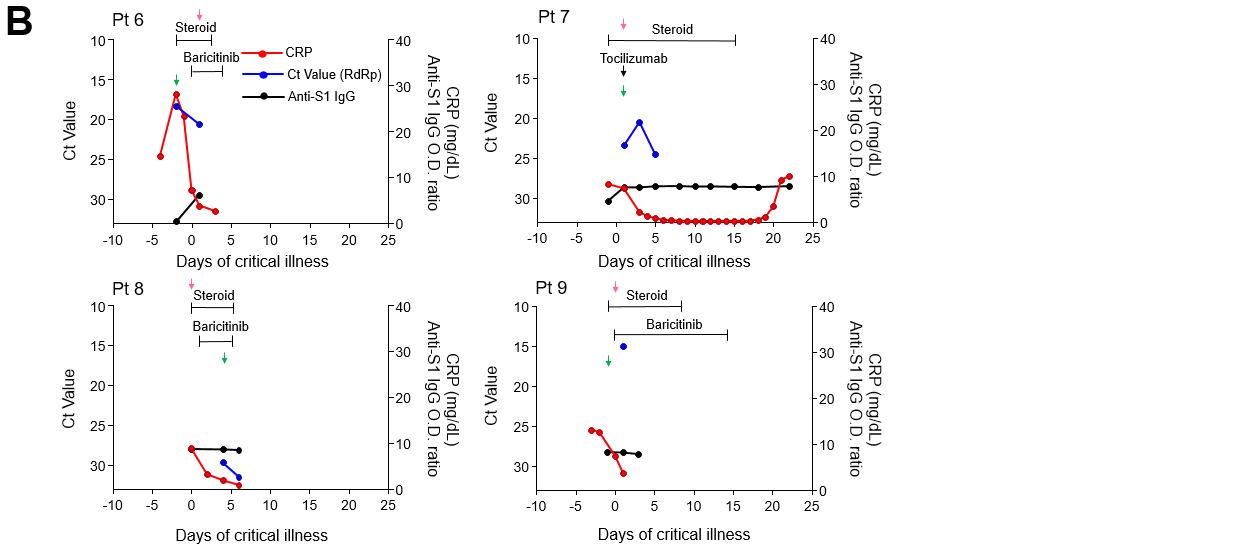
**


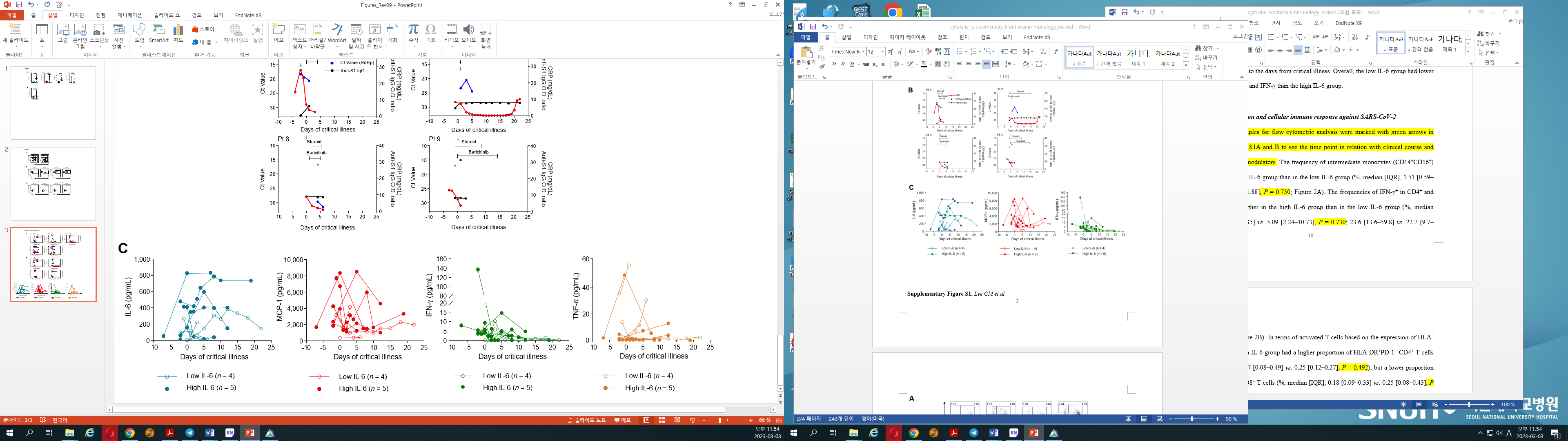


**Supplementary Figure S1.** *Lee CM et al.*

**
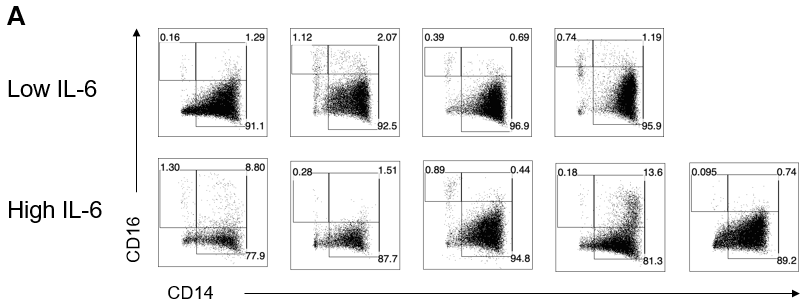
**

**
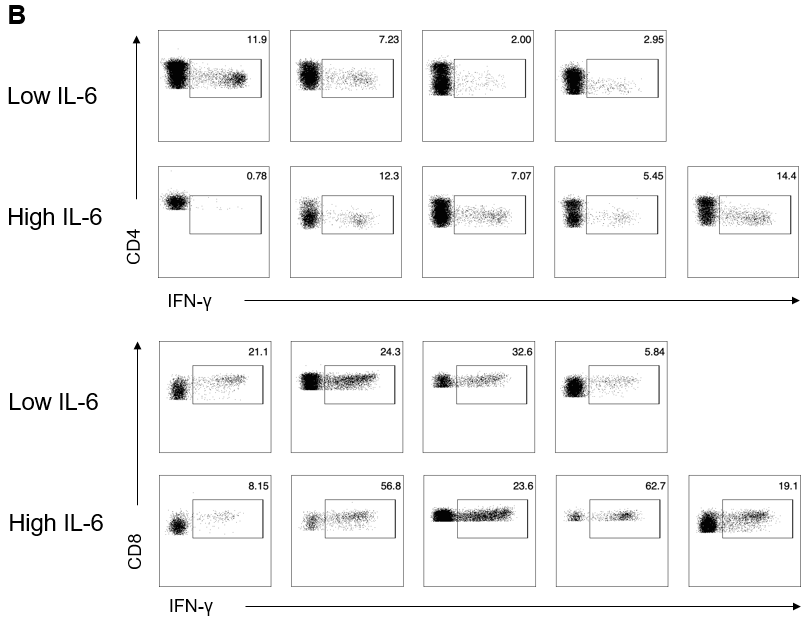
**

**
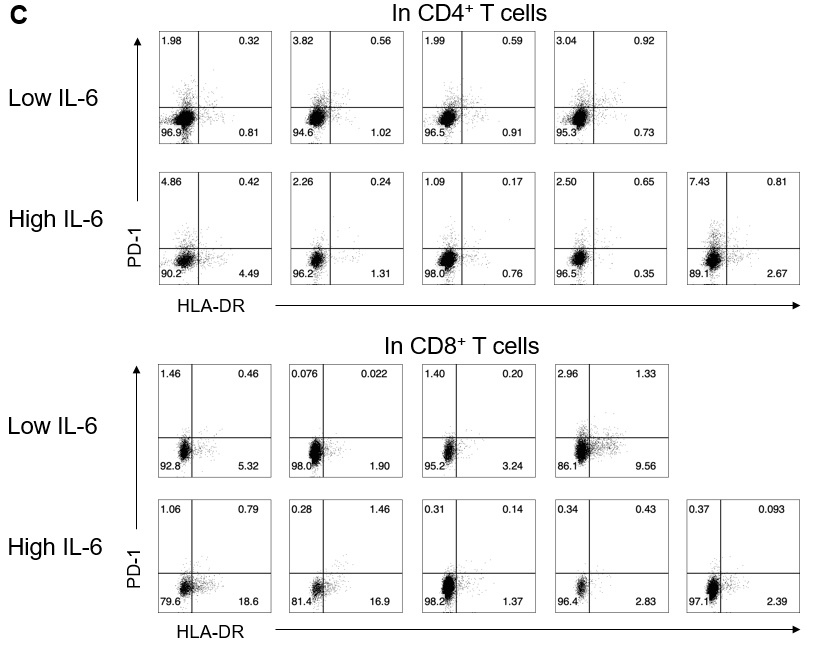
**

**Supplementary Figure S2.** *Lee CM et al.*
